# Precocious puberty in children: A review of imaging findings

**DOI:** 10.2349/biij.8.1.e6

**Published:** 2012-01-01

**Authors:** MZ Faizah, AH Zuhanis, R Rahmah, AA Raja, LL Wu, AA Dayang, MA Zulfiqar

**Affiliations:** 1 Department of Radiology, Universiti Kebangsaan Malaysia Medical Centre (UKMMC), Kuala Lumpur, Malaysia; 2 Department of Paediatrics, Universiti Kebangsaan Malaysia Medical Centre (UKMMC), Kuala Lumpur, Malaysia; 3 Paediatric Surgical Unit, Department of Surgery, Universiti Kebangsaan Malaysia Medical Centre (UKMMC), Kuala Lumpur, Malaysia

**Keywords:** precocious puberty, imaging

## Abstract

**Objectives:**

This review was aimed at determining the imaging findings in patients with precocious puberty.

**Results:**

Within a period of 8 years (from 2002 to 2010) there were 53 patients diagnosed with precocious puberty. Out of the 53 patients, 37 had undergone diagnostic imaging to detect the possible organic causes of precocious puberty. Imaging findings were positive in 31 patients and out of that, 3 patients had 2 findings each (34 abnormalities). Of the patients with positive imaging findings, central precocious puberty (gonadotrophin-dependent) was more common (81%; 25/31) and the causes included: tuber cinereum hamartoma (n = 10), glioma (n = 6), pineal gland tumour (n = 4), hydrocephalous (n = 3), arachnoid cyst (n = 2) and others (n = 3). Peripheral precocious puberty (gonadotrophin-independent) causes included: testicular adrenal rest tumour (n = 3), adrenal carcinoma (n = 1), ovarian granulosa thecal cell tumour (n = 1), and tuberous sclerosis (n = 1).

**Conclusion:**

Positive imaging findings were observed in 84% (31/37) of the subjects. Hypothalamic hamartoma was the most common imaging finding in central precocious puberty while testicular adrenal rest tumour was the most common imaging finding in peripheral precocious puberty.

## INTRODUCTION

Precocious puberty is defined as the development of secondary sexual characteristics before the age of 8 years in girls and 9 years in boys [1–3]. Two types of precocious puberty are recognised, central precocious puberty (CPP) and peripheral precocious puberty (PPP) [1–3].

In order to understand the two types of precocious puberty, basic knowledge of normal puberty pertaining to the hypothalamus-pituitary-gonadal (HPG) axis is necessary [2, 3]. The gonadotrophin-releasing hormone (GnRH) that is produced by the brain’s hypothalamus leads to activation of the anterior pituitary to produce and release gonadotrophins, luteinising hormone (LH), and follicle-stimulating hormone (FSH). These hormones will then activate the gonads (testes in boys and ovaries in girls) to produce the male sex hormone (testosterone) and the female sex hormone (oestrogen), respectively. The hormones produced by the gonads lead to the physical and sexual changes of puberty [2, 3].

CPP, which is also called GnRH-dependent precocious puberty, is caused by early activation of the HPG axis [1, 3]. It is more common in girls, and is usually idiopathic. Secondary causes of CPP includes: brain tumours, brain infections, congenital brain defects, radiation or injury to the brain or spinal cord, and brain ischaemia. The most common cause of CPP is hypothalamic tumour, in particular the tuber cinereum hamartoma followed by hydrocephalus and previous central nervous system (CNS) injury [1].

PPP, which is also called GnRH-independent precocious puberty, does not involve the HPG axis. It is caused by release of oestrogen or testosterone into the body from abnormal organs and causes include adrenal hyperplasia, adrenal tumour, and gonadal tumour [1].

This review was aimed at determining the imaging findings in patients with precocious puberty.

## MATERIALS AND METHODS

This was a retrospective review of the radiological findings of patients diagnosed with precocious puberty. The list of patients with precocious puberty from 2002 until 2010 was obtained from the paediatric endocrinologist at the medical centre where this study was carried out. Based on this list, the radiological reports of patients who had undergone radiological investigations were reviewed. These reports were retrieved from the Integrated Radiology Information System (IRIS). The radiological images were reviewed from the Picture Archiving and Communication System (PACS) (Medweb®) for patients imaged after September 2007. Hardcopy images were reviewed for patients imaged prior to September 2007.

**Figure 1:**
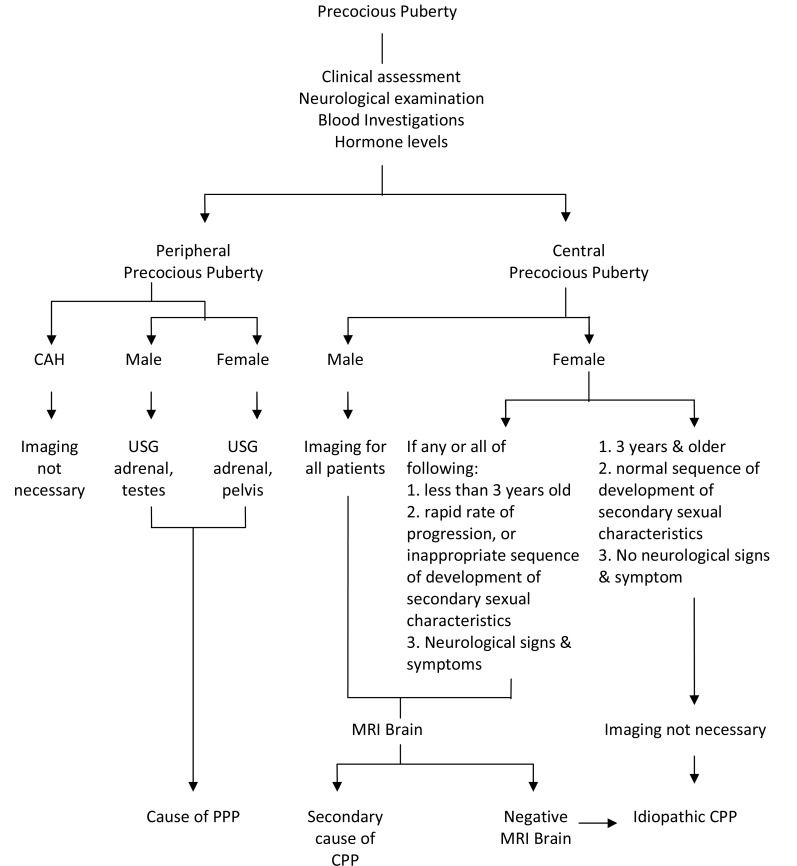
Imaging algorithm for precocious puberty.

The diagnosis of precocious puberty was made based on clinical evaluation, plasma hormonal investigations (LH, FSH and testosterone/ oestradiol levels), and assessment of bone age by the paediatric endocrinologist. Radiological investigations were performed when indicated (Figure 1). The decision to perform radiological investigations was made by the paediatric endocrinologist based on several factors which included: very young age (below 3 years old), rapid progression of puberty, presence of neurological signs and symptoms, and biochemical investigations that point towards pathological precocious puberty. The senior medical officer or radiologist performed the ultrasound scans (USG) using ultrasound machine (HD11 or IU22, Philips, Eindhoven,The Netherlands). Computed tomography (CT) with contrast was performed in axial plane with multi-planar reconstruction (Siemens SOMATOM 64-slices, Erlangen, Germany). The MRI was performed using two different machines; prior to year 2006 (Siemens MAGNETOM 1.5T, Erlangen, Germany) and after year 2006 (Siemens AVANTO 1.5T, Erlangen, Germany).

Data were recorded in Microsoft Excel and were analysed using the statistical packages SPSS (version 16) for its descriptive statistics. Statistical analysis was not performed, as this is a descriptive evaluation.

## RESULTS

From 2002 until 2010, there was a total of 53 patients (35 girls and18 boys) aged between 1 and 15 years (mean age of 6.9 years) with a diagnosis of precocious puberty. Some had been diagnosed before the study period (before 2002) and therefore children older than age 8 and 9 years were included in the sample.

Out of 53 patients reviewed, 70% (37/53) had undergone some form of radiological investigation: MRI of the brain, ultrasound of the pelvis/testes or CT of the abdomen. Out of the 37 patients who underwent radiological investigations, 84% (31/37) had abnormal findings. Three of the patients had 2 findings each. These were concomitant intracranial tumour with hydrocephalus. This resulted in a total of 34 abnormalities. Six patients had normal MRI of the brain and were therefore diagnosed with idiopathic CPP. This was more common in girls (n = 5) compared to boys (n = 1). Of the 16 patients who were not imaged, 11 were girls diagnosed with idiopathic CPP, while 4 girls and 1 boy were diagnosed with congenital adrenal hyperplasia.

**Figure 2:**
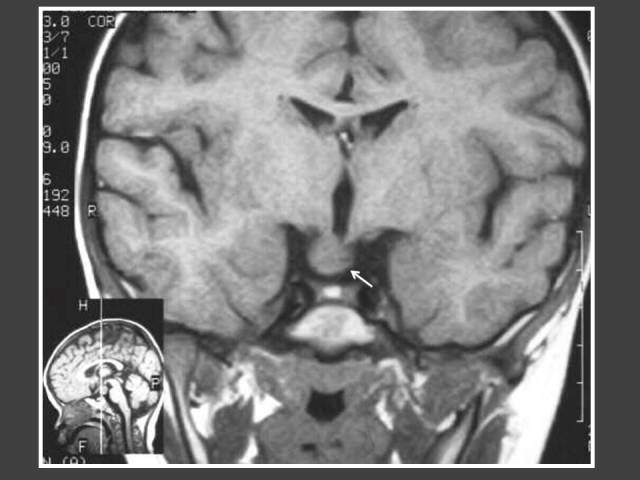
One year-old boy who presented with penile enlargement. Coronal T1WI MRI of the brain shows mass in hypothalamus (arrow) representing tuber cinerium hamartoma.

**Figure 3:**
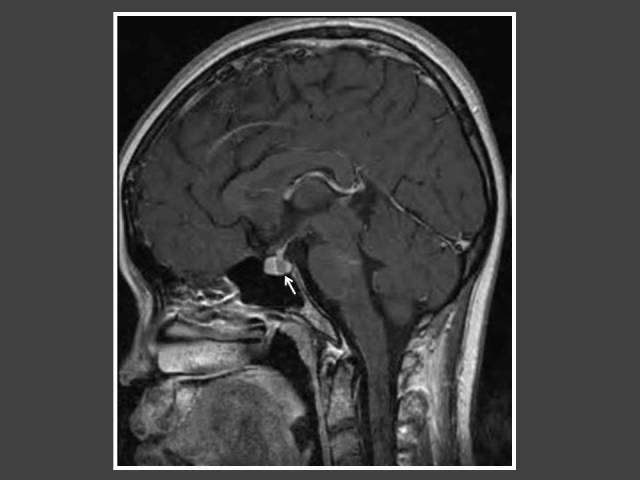
A girl with global developmental delay who presented with bilateral breast enlargement. T1WI Sagittal MRI of the brain (post-gadolinium dynamic protocol) shows an intrasellar lesion representing pituitary microadenoma (arrow).

**Figure 4:**
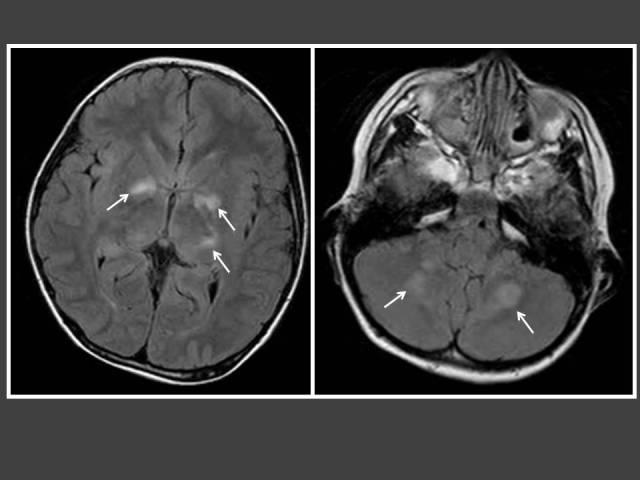
Seven year-old boy with neurofibromatosis Type 1 (NF-1) who presented with hirsutism. T2-FLAIR MRI of the brain shows multiple foci of high signal intensity in the basal ganglia and cerebelli (arrows) consistent with myelin vacuolation.

**Figure 5:**
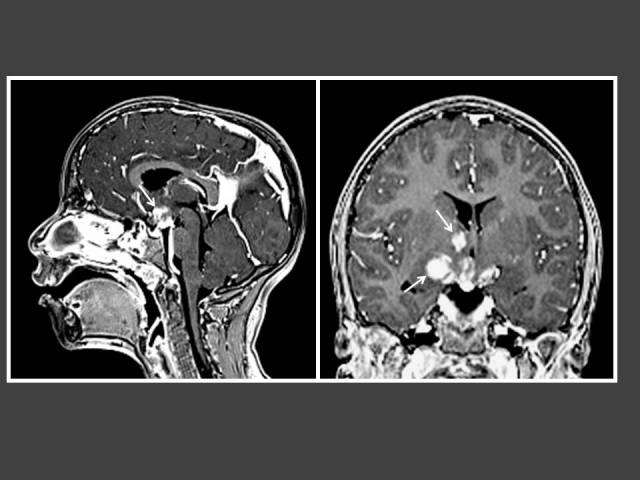
Five year-old boy who presented with penile enlargement. T1WI post-gadolinium MRI of the brain in sagittal and coronal views demonstrates multiple enhancing lesions in the hypothalamus and right basal ganglia (arrows). Histology showed intracranial germ cell tumour.

There were 82% (28/34) GnRH-dependent causes detected on imaging as compared to 18% (6/34) GnRH-independent causes. Among the girls with CPP, 45% (13/29) had intracranial pathology and 55% (16/29) were diagnosed as idiopathic CPP (Table 1). Among the boys with CPP, 92% (12/13) had intracranial pathology while 8% (1/13) had idiopathic CPP (Table 1). The GnRH-dependent causes seen on imaging included: hypothalamic hamartoma (n = 10), hydrocephalus (n = 3), astrocytoma/glioma (n = 6), pineal gland tumour (n = 4), arachnoid cyst (n = 2) and 1 case each for pituitary microadenoma, myelin vacuolation in neurofibromatosis Type 1 (NF-1) and suprasellar germ cell tumour with cerebral metastases (Figures 2–5).

**Table 1:** Causes of precocious puberty.

	**Causes of CPP**	**Girls**	**Boys**	**Total**
1	idiopathic	16	1	17
2	hamartoma	6	4	10
3	astro/glioma	3	3	6
4	pineal gland tumour	2	2	4
5	hydrocephalus^##^	1^##^	2^##^	3^##^
6	arachnoid cyst	1	1	2
7	intra-cranial germinoma	0	1	1
8	pituitary macroadenoma	1	0	1
9	neurofibromatosis Type 1	0	1	1
	**Total number of patients with CPP**	**29**	**13**	**42**
	**Causes of PPP**			
1	congenital adrenal hyperplasia	4	1	5
2	testicular adrenal rest tumour	0	3	3
3	adrenal carcinoma	0	1	1
4	ovarian granulosa cell tumour	1	0	1
5	tuberous sclerosis	1	0	1
	**Total number of patients with PPP**	**6**	**5**	**11**
	**Total with PP**	**35**	**18**	**53**

Key: ^##^ Hydrocephalus was an associated finding in patients with intracranial tumour

**Figure 6:**
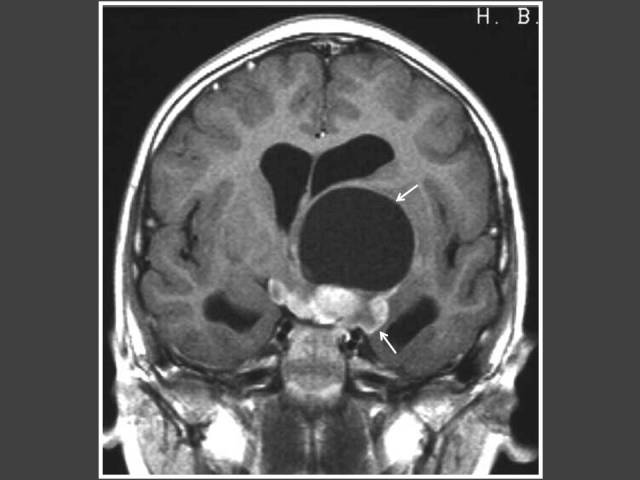
Four year-old boy who presented with impaired vision and acne. T1WI post-gadolinium sagittal MRI of the brain shows heterogenously enhancing suprasellar tumour (arrows) with hydrocephalus. Histology revealed pilomyxoid astrocytoma.

**Figure 7:**
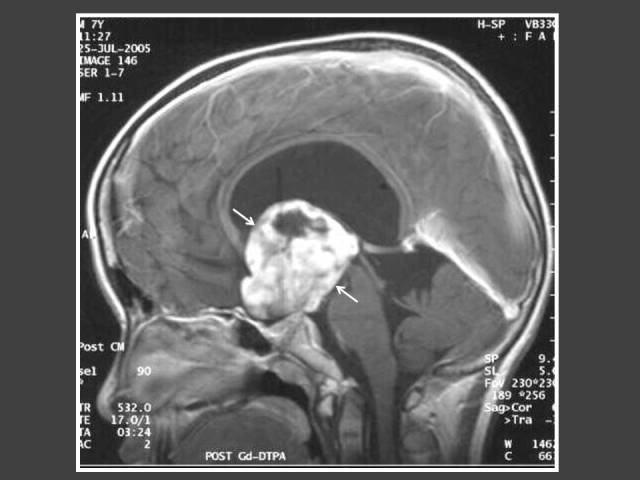
Eight year-old boy who presented with signs of increased intracranial pressure and hirsutism. MRI shows heterogenously enhancing suprasellar tumour (arrows) with hydrocephalus. Histology showed low-grade astrocytoma.

**Figure 8:**
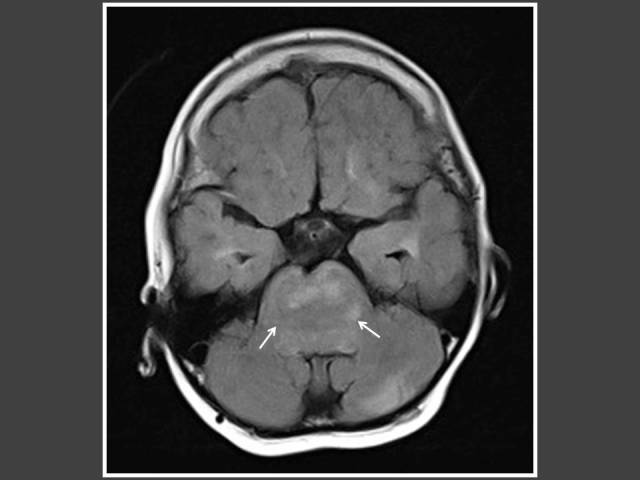
Seven years-old girl who presented with bilateral breast enlargement. T2-FLAIR axial MRI of the brain shows brainstem glioma (arrows).

**Figure 9:**
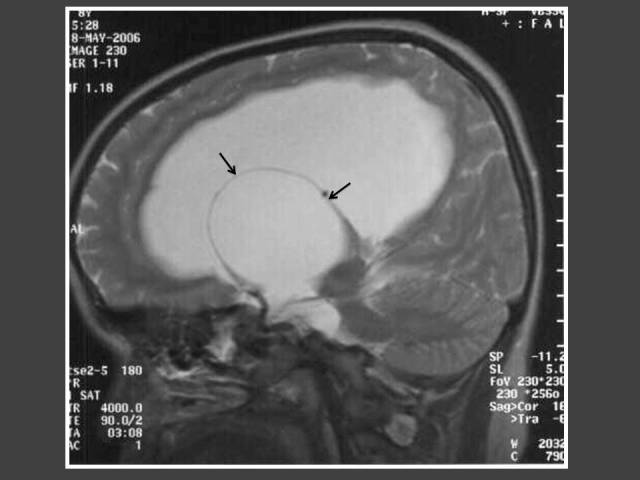
Eight year-old girl who presented with bilateral breast enlargement. T2WI sagittal MRI of the brain shows suprasellar arachnoid cyst (arrows) with hydrocephalus.

**Figure 10:**
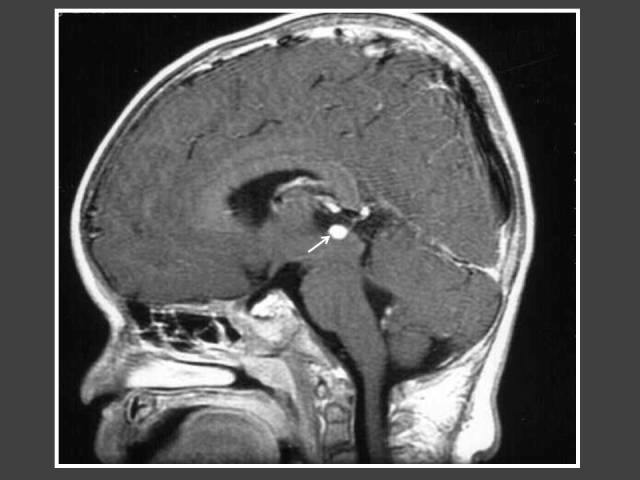
Three year-old boy with increased penile size. T1WI post-gadolinium sagittal MRI of the brain shows an enhancing pineal gland tumour (arrow). Histology was consistent with germinoma.

The most common cause of intracranial tumour in CPP was hypothalamic hamartoma, which was true in both male and female populations. In 4 out of 6 patients with astrocytoma/glioma cases, the location of the tumour was in the supratentorial region. Two of these cases had concomitant hydrocephalus (Figures 6 and 7). The other 2 glioma cases occurred in girls and were found in the brainstem (Figure 8). Both did not have neurofibromatosis. One of the two arachnoid cysts was located in the suprasellar region and complicated with hydrocephalus (Figure 9) while the other was located in the left middle cranial fossa. There were 4 pineal gland tumours which consisted of pineal gland germinoma (n = 2) (Figure 10), pineocytoma (n = 1) and pineal cyst (n = 1).

**Figure 11:**
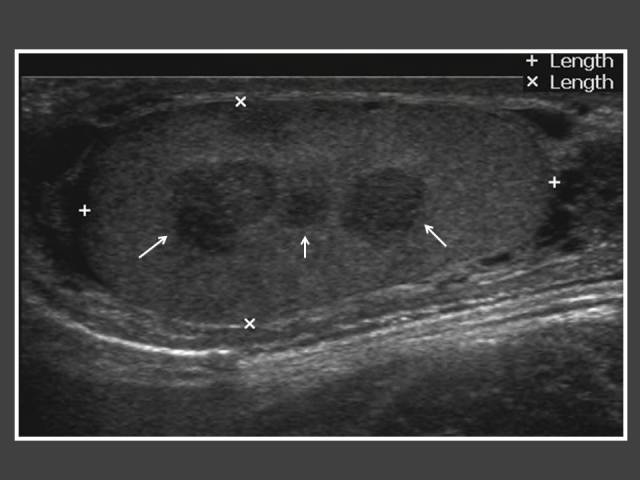
Twelve year-old boy who was diagnosed earlier with congenital adrenal hyperplasia at the age of 2 and was on steroid treatment. An ultrasound of the testis was performed because of resistance to steroid therapy. It shows multiple hypoechoic lesions within the testis (arrows) representing testicular adrenal rest tumour.

**Figure 12:**
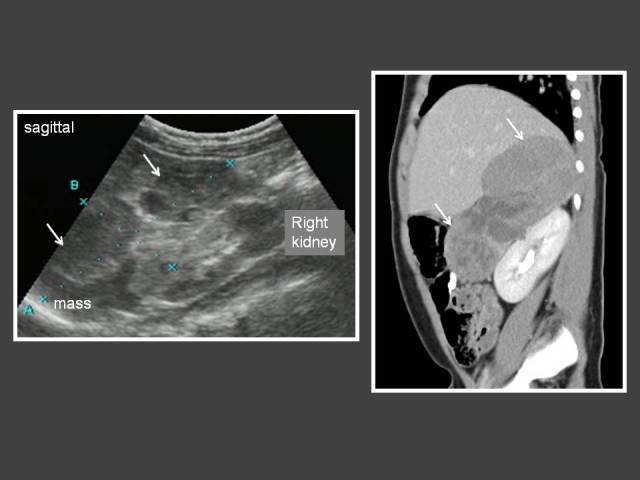
Two year-old boy who presented with hirsutism and acne. An ultrasound and subsequent CT (sagittal reconstruction of contrast-enhanced CT abdomen) shows a heterogenous mass in the right suprarenal region (arrows). There is no calcification within the mass. The tumour was surgically removed and histology revealed adrenocortical carcinoma. The child responded well to chemotherapy.

**Figure 13:**
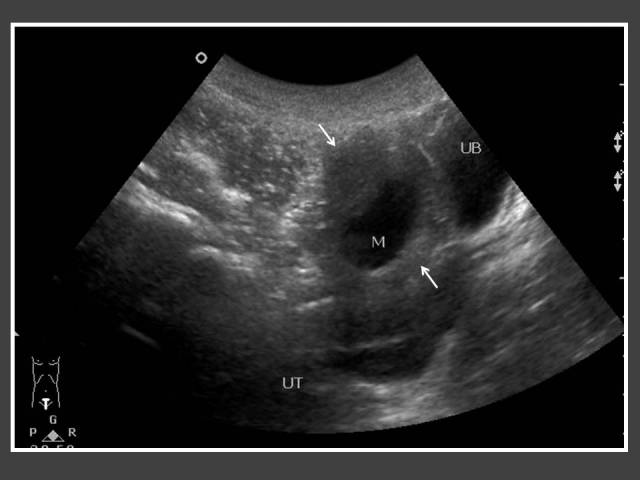
Four year-old girl who presented with per-vaginal bleeding. Transverse view of the pelvic ultrasound shows a right ovarian cyst with thickened walls (arrows). She underwent laparoscopic removal of the ovarian cyst and the histology revealed a granulosa thecal cell tumour.

There were 8 patients with congenital adrenal hyperplasia (CAH) in our review. Three had imaging studies performed because they were resistant to steroid therapy. All 3 cases showed testicular adrenal rest tumour (TART) (Figure 11). Other GnRH-independent PP were adrenocortical carcinoma (n = 1) (Figure 12), ovarian granulosa thecal cell tumour (n = 1) (Figure 13) and tuberous sclerosis (n = 1). The CT abdomen of the child with tuberous sclerosis showed bilateral angiomyolipoma.

## DISCUSSION

Precocious puberty is caused by a heterogeneous group of disorders, which ranges from idiopathic to malignant tumours [1]. There are several causes of premature sexual development which can be divided into: i) premature activation of the hypothalamic-pituitary-gonadal (HPG) axis (central PP); ii) abnormal patterns of gonadotrophin secretion (premature thelarche, thelarche variant); iii) excess adrenal androgens (adrenarche, congenital adrenal hyperplasia (CAH), adrenal tumours); and iv) gonadotrophin independent PP (secretion of sex steroids is independent of the HPG axis) [2]. The patients reviewed in this series were mostly in the first and fourth category.

Premature thelarche or thelarche variant is characterised by isolated premature breast development and is associated with normal growth velocity and bone age advancement within two standard deviations of normal. It is thought to be a self-limiting condition likely to resolve within 6 months to 6 years after diagnosis [2].

### Central precocious puberty (CPP)

Central precocious puberty or GnRH-dependent precocious puberty is more common by far in girls than in boys, where in girls it is usually idiopathic. Central nervous system disorders account for a higher percentage of cases in boys but must also be excluded in girls [2, 4]. Approximately 95% of girls with CPP have idiopathic CPP and only 5% have a secondary cause. Whereas more than 50% of boys have an identifiable aetiology and idiopathic CPP is a diagnosis of exclusion [2]. We observed a much higher percentage for a secondary cause of CPP of 45% (13/29) in our female population. MRI of the brain was not routinely done in girls with CPP at this centre (Figure 1). Therefore, the true incidence of a secondary cause of CPP could be higher. It has been advocated that girls with CPP should have a cranial MRI as part of their assessment since clinical features, including age, are not helpful in predicting those with underlying pathology [4]. This is the reverse for CPP in boys whereby most of the boys have a secondary cause of CPP with the main cause being a central nervous system tumour. Therefore, CNS disease must first be ruled out before diagnosing a patient as having idiopathic precocious puberty [5]. At this centre, all males with precocious puberty were imaged. Imaging was not routinely done in girls with CPP. This review showed that almost all (92%) males with CPP had intracranial pathology.

The most common underlying disorders include tumours of the hypothalamic region, especially hamartoma of the tuber cinereum, hydrocephalus and previous central nervous system (CNS) injury from any cause [1]. The prevalence of intracranial pathology in this review was 47% (25/53). This is similar to previous reports, which demonstrated prevalence of intracranial pathology of 15% to 49% [4, 6–8]. Several studies have reported on the incidence of hypothalamic hamartomas in patients with precocious puberty, varying from 14% to 58% [4]. In a review of MRI findings and clinical features, 8 out of 9 patients with hypothalamic hamartoma had precocious puberty [9]. In this review, it was also found that hypothalamic hamartoma was the most common tumour causing CPP in the patients, accounting for 10 out of 34 of the abnormal imaging findings (29%). The aim of treatment is to preserve final height and reverse physical changes to pre-pubertal stage congruous to chronological age. For these patients, the left hand and wrist radiograph was used to monitor bone age. Of the 10 cases of hamartoma detected in this series, the indications for imaging included: 8 with early onset of precocious puberty before the age of 3 years, and 4 male patients.

Precocious puberty and amenorrhoea have been associated with hydrocephalus. The exact pathway by which hydrocephalus disrupts the hypothalamic GnRH system is unknown. However, previous reports postulated that compressive forces, ischaemia, and impairment of the neurotransmitter feedback loop might be the explanation [1]. We had 3 cases of CPP due to hydrocephalus, which occurred as a complication of suprasellar tumour.

Most tumours of the chiasm and hypothalamus in children are gliomas and the majority are low grade at histology [11]. The authors noted that most of the non-hypothalamic intracranial tumours causing CPP were located in the suprasellar region with the pineal gland being the second most common location. All of the patients in this study with astrocytoma had the tumour in the suprasellar region.

Brainstem gliomas are the second most frequent tumour in NF-1 after optic tract tumour [12]. Brainstem glioma presenting with precocious puberty has been reported in patients with NF-1. In this review, of the 2 cases of brainstem glioma, one had histology of glioblastoma multiforme which later recurred. Neither case has NF-1.

Arachnoid cysts are relatively uncommon intracranial lesions, usually developmental in origin [13]. The majority occur in the supratentorial compartment and, of these, roughly 9–15% occur in the suprasellar region [11].

It is known that tumours and other pathological processes involving the hypothalamus frequently modify sexual development. These lesions may destroy the posterior hypothalamus, leaving the anterior hypothalamus intact, which leads to increased pituitary function and hence, causes CPP [14]. This also explains how CPP occurs when suprasellar tumours such as astrocytoma, arachnoid cyst or germ cell tumours compress upon the posterior hypothalamus due to the close proximity of the hypothalamus.

Germ cell tumours most frequently arise in the pineal and suprasellar region and, in general, pineal gland germ cell tumours outnumber suprasellar tumours by a ratio of 2:1 [15]. The most common pineal tumours are germ cell tumours, besides pineal parenchymal tumours or astrocytomas [16]. The authors observed 2 germ cell tumours originating from the pineal gland and 1 suprasellar germ cell tumour with intracerebral metastases.

A previous study had shown that CPP occurred in 3% of their entire population of children with NF-1, which is markedly higher than its incidence in the general population (0.06%) [17]. CPP was found exclusively in those children with NF-1 who had optic pathway tumours (OPTs) involving the optic chiasm [17, 18]. Therefore, it is peculiar that our only patient with NF-1 had myelin vacuolation without OPTs but still presented with CPP. Two NF-1 cases who presented with CPP but without OPTs have been reported. It was theorised that the temporary neurologic manifestations of NF-1 may be sufficient to alter the hypothalamus-pituitary set point for triggering puberty [18].

### Peripheral precocious puberty (PPP)

In peripheral precocious puberty or GnRH-independent precocious puberty, boys develop secondary sexual characteristics from two conditions: secretion of androgens from the testes or adrenal glands, or rarely, secretion of human chorionic gonadotropin (hCG) or LH, which can stimulate Leydig cell production of testosterone [1]. Congenital adrenal hyperplasia (CAH) is the most common cause of PPP [1]. On the other hand, rarer causes would include tumours that produce hCG, testicular or adrenal tumours that produce testosterone, and McCune–Albright syndrome [19].

There are some patients with unrecognised or poorly-controlled congenital adrenal hyperplasia who present with testicular masses consisting of adrenal rest tissue [20]. Testicular adrenal rest tumours (TART) in males showed variable prevalence between 0 and 94%, depending on the selection of patients (age, hormonal control) and the method of tumour detection [21]. Ultrasound and MRI are equally good methods for detection and monitoring of the tumours, but ultrasound is preferable because it is quick and cheap [21]. The authors had performed adrenal and testicular ultrasound in patients who were resistant to CAH therapy and found 3 TART cases. Two of the cases were subjected to testicular sparing surgery in order to control the CAH.

The most common cause of PPP in girls is ovarian tumour. It affects 11% of all girls with PPP and granulosa thecal cell tumour is the most common type [5]. PPP in girls is due to excessive oestrogen that can cause breast development and even vaginal bleeding [1].

A rare cause of precocious puberty in our review was a case of tuberous sclerosis (TS). TS is associated with a number of abnormalities in endocrine tissues, where the adrenal gland is the most frequent endocrine gland affected. Endocrine abnormalities in patients with TS include precocious puberty, which may be due to gonadal activation by the pituitary gland or by gonadotrophin-independent mechanisms [22].

Other rare causes of PPP seen in both sexes that were not present in this review include McCune Albright syndrome, severe hypothyroidism, and iatrogenic or exogenous sexual precocity [1].

## CONCLUSION

This review demonstrated that positive imaging findings were observed in 84% of cases and secondary cause of CPP in girls was higher than previously reported. It is not necessary to image all girls with CPP as there is still a place for clinical assessment. Imaging is mandatory in all boys with CPP. Hypothalamic hamartoma was found to be the most common finding on imaging in CPP while testicular adrenal rest tumour was the most common finding on imaging in PPP.
